# Crystal structure of *cis*-di­chlorido­(1,4,8,11-tetra­aza­cyclo­tetra­decane-κ^4^ *N*)chromium(III) (oxalato-κ^2^ *O* ^1^,*O* ^2^)(1,4,8,11-tetra­aza­cyclo­tetra­decane-κ^4^ *N*)chromium(III) bis(perchlorate) from synchrotron data

**DOI:** 10.1107/S2056989016014134

**Published:** 2016-09-09

**Authors:** Dohyun Moon, Jong-Ha Choi

**Affiliations:** a Pohang Accelerator Laboratory, POSTECH, Pohang 37673, Republic of Korea; b Department of Chemistry, Andong National University, Andong 36729, Republic of Korea

**Keywords:** crystal structure, cyclam, synchrotron radiation, chromium(III) complex, chloride ligand, oxalato ligand, *cis*-V conformation, hydrogen bonding

## Abstract

In the asymmetric unit of the title compound, [CrCl_2_(C_10_H_24_N_4_)][Cr(C_2_O_4_)(C_10_H_24_N_4_)](ClO_4_)_2_ (C_10_H_24_N_4_ = 1,4,8,11-tetra­aza­cyclo­tetra­decane, cyclam; C_2_O_4_ = oxalate, ox), there are two independent halves of the [CrCl_2_(cyclam)]^+^ and [Cr(ox)(cyclam)]^+^ cations, and one perchlorate anion. In the complex cations, which are completed by application of twofold rotation symmetry, the Cr^III^ ions are coordinated by the four N atoms of a cyclam ligand, and by two chloride ions or one oxalate bidentate ligand in a *cis* arrangement, displaying an overall distorted octa­hedral coordination environment. The Cr—N(cyclam) bond lengths are in the range of 2.075 (5) to 2.096 (4) Å while the Cr—Cl and Cr—O(ox) bond lengths are 2.3358 (14) and 1.956 (4) Å, respectively. Both cyclam moieties adopt the *cis*-V conformation. The slightly distorted tetra­hedral ClO_4_^−^ anion remains outside the coordination sphere. The supra­molecular architecture includes N—H⋯O and N—H⋯Cl hydrogen bonding between cyclam NH donor groups, O atoms of the oxalate ligand or ClO_4_^−^ anions and one Cl ligand as acceptors, leading to a three-dimensional network structure.

## Abstract 2

Two Cr^III^ ions (each with site symmetry 2..) in the title compound have a distorted octa­hedral coordination environment with four N atoms of a cyclam ligands and two chloride ions or one oxalate bidentate ligand in a *cis* position whereby the cyclam ligands adopt a *cis*-V conformation. The crystal packing is stabilized by extensive hydrogen-bonding inter­actions among the cyclam N–H groups, the Cl ligands, and O atoms of the oxalate and ClO_4_^−^ anions.

## Chemical context   

**Figure 1:**
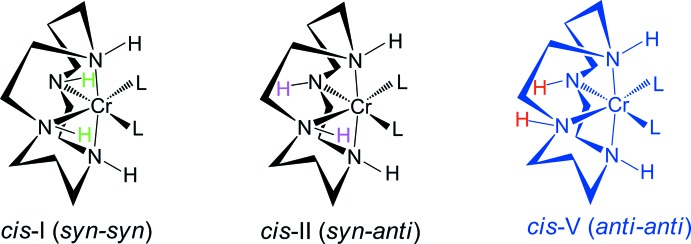
Possible conformers of *cis*-[Cr*L*_2_(cyclam)]^n+^ complexes.

Transition metal complexes with cyclam (1,4,8,11-tetra­aza­cyclo­tetra­decane, C_10_H_24_N_4_) ligands can adopt both planar (*trans*) and folded (*cis*) configurations (Poon & Pun, 1980 ▸). The possible conformers of the *trans* isomer are *trans*-I (+ + + +), *trans*-II (+ – + +), *trans*-III (+ – – +) and *trans*-V (+ + – –), which differ in the chirality of the *sec*-NH groups (Choi, 2009 ▸) and where + indicates if the H atom of the NH group is above the plane of the macrocycle and – indicates if it is below. The *trans*-I, *trans*-II and *trans*-V conformations can fold to form *cis*-I, *cis*-II and *cis*-V conformers, as shown in Fig. 1 ▸. The *trans*-III conformation gives the most thermodynamically stable complex with two six-membered rings in chair and two five-membered rings in *gauche* conformations (Choi, 2009 ▸). However, the most stable conformation cannot fold to give the *cis-*III complex as this requires the diagonal NH groups to both lie above or below the plane of the macrocycle.

Recently, it has been shown that cyclam derivatives and their metal complexes exhibit anti-HIV activity (Ronconi & Sadler, 2007 ▸; De Clercq, 2010 ▸; Ross *et al.*, 2012 ▸). The conformation of the macrocyclic ligand and the orientations of the N—H bonds are very important factors for co-receptor recognition. Therefore, knowledge of the conformation and crystal packing of transition metal complexes containing the cyclam ligand has become important in the development of new highly effective anti*-*HIV drugs that specially target alternative events in the HIV replicative cycle (De Clercq, 2010 ▸).

In this communication, we report on the synthesis and structural characterization of a new double complex, [CrCl_2_(cyclam)][Cr(ox)(cyclam)](ClO_4_)_2_, (I).
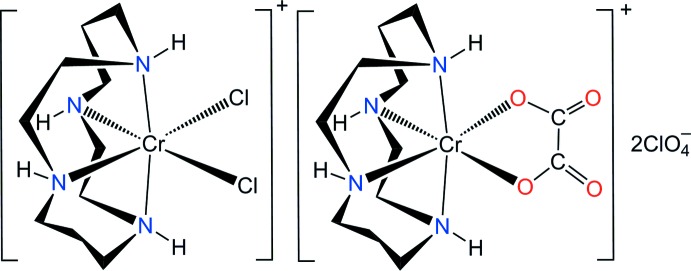


## Structural commentary   

The asymmetric unit contains two halves of the [CrCl_2_(cyclam)]^+^ and [Cr(ox)(cyclam)]^+^ cations, and one perchlorate anion. Each cyclam moiety exhibits point group symmetry ..2 and can be described as being in the *cis*-V (*anti*–*anti*) conformation (Fig. 1 ▸). In each complex cation, the Cr^III^ ions are coordinated by the N atoms of the cyclam ligands; two oxygen atoms of the oxalato ligand for one and two chlorido ligands for the other cation complete distorted octa­hedral coordination spheres binding their N atoms in a *cis* configuration (Fig. 1 ▸). The Cr—N bond lengths from the donor atoms of the cyclam ligands are in the range of 2.075 (5) to 2.096 (4) Å, in good agreement with those determined in *cis-*[Cr(N_3_)_2_(cyclam)]ClO_4_ [2.069 (3)–2.103 (3) Å] (Meyer *et al.*, 1998 ▸), *cis*-[Cr(ONO)_2_(cyclam)]NO_2_ [2.0874 (16)–2.0916 (15) Å] (Choi *et al.*, 2004*a* ▸), [Cr(acac)(cyclam)](ClO_4_)_2_·0.5H_2_O [2.070 (5)–2.089 (5) Å] (acac = acetyl­acetonate; Subhan *et al.*, 2011 ▸) and *cis*-[Cr(NCS)_2_(cyclam)]NCS [2.0851 (14)–2.0897 (14) Å] (Moon *et al.*, 2013 ▸). However, the Cr—N bond lengths of the cyclam ligand in the *cis* conformation are slightly longer than those found in *trans-*[Cr(NCS)_2_(cyclam)]ClO_4_ [2.046 (2)–2.060 (2) Å] (Friesen *et al.*, 1997 ▸), *trans*-[Cr(ONO)_2_(cyclam)]BF_4_ [2.064 (4)–2.073 (4) Å] (De Leo *et al.*, 2000 ▸), *trans-*[Cr(NH_3_)_2_(cyclam)][ZnCl_4_]Cl·H_2_O [2.0501 (15)–2.0615 (15) Å] (Moon & Choi, 2016 ▸) and *trans*-[Cr(nic-O)_2_(cyclam)]ClO_4_ [2.058 (4)–2.064 (4) Å] (nic-O = O-coordinated nicotinate; Choi, 2009 ▸). The Cr—N bond lengths of the secondary amine are also comparable to those involving the primary amine found in *trans*-[CrCl_2_(Me_2_tn)_2_]_2_ZnCl_4_ (Me_2_tn = 2,2-di­methyl­propane-1,3-di­amine; Choi *et al.*, 2011 ▸), *trans*-[Cr(N_3_)_2_(Me_2_tn)_2_]ClO_4_·2H_2_O (Moon & Choi, 2015 ▸), *trans*-[Cr(NCS)_2_(Me_2_tn)_2_]SCN·0.5H_2_O (Choi & Lee, 2009 ▸) and *trans-*[Cr(2,2,3-tet)F_2_]ClO_4_ (2,2,3-tet = 1,4,7,11-tetra­aza­undecane; Choi & Moon, 2014 ▸). The Cr1*A*—O1*A* bond length of 1.956 (4) Å for the oxalate ligand is close to the mean of 1.959 (4) Å found in [Cr(ox)(cyclam)]ClO_4_ (Choi *et al.*, 2004*b* ▸). The Cr1*B*—Cl1*B* bond length of 2.3358 (14) Å is comparable to those found in *cis-*[CrCl_2_(cyclam)]ClO_4_ [2.331 (2) Å] (House & McKee, 1984 ▸), *cis-*[CrCl_2_(2,2,3-tet)]ClO_4_ [2.3157 (7) Å] (Choi *et al.*, 2008 ▸), *trans*-[CrCl_2_(Me_2_tn)_2_]_2_ZnCl_4_ [2.3112 (6) Å] (Choi *et al.*, 2011 ▸) and *trans*-[CrCl_2_(Me_2_tn)_2_]Cl [2.3253 (7) Å] (Choi *et al.*, 2007 ▸), respectively. The five-membered and six-membered chelate rings of the cyclam ligands adopt *gauche* and stable chair conformations, respectively. The O1*A*—Cr1*A*—O1*A* ^i^ angle is 83.3 (3)°, while the Cl1*B*—Cr1*B*—Cl*B* ^i^ angle is 89.11 (9)° [symmetry code: (i) –*x* + 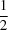, −*y* + 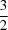, *z*]. The folded angles of the cyclam in [CrCl_2_(cyclam)]^+^ and [Cr(ox)(cyclam)]^+^ cations are 93.7 (2) and 97.5 (2)°, respectively. The significant distortion of the octa­hedral coordination sphere and the larger folded angle in the [Cr(ox)(cyclam)] ^+^ cation seem to arise from the small bite angle of the oxalato ligand. The tetra­hedral ClO_4_^−^ anion remains outside the coordination sphere of two Cr^III^ ions. It is distorted due to its involvement in hydrogen-bonding inter­actions. Cl—O bond lengths range from 1.426 (5) to 1.443 (5) Å and the O—Cl—O angles from 107.8 (4)–111.0 (3)°.

## Supra­molecular features   

**Figure 2:**
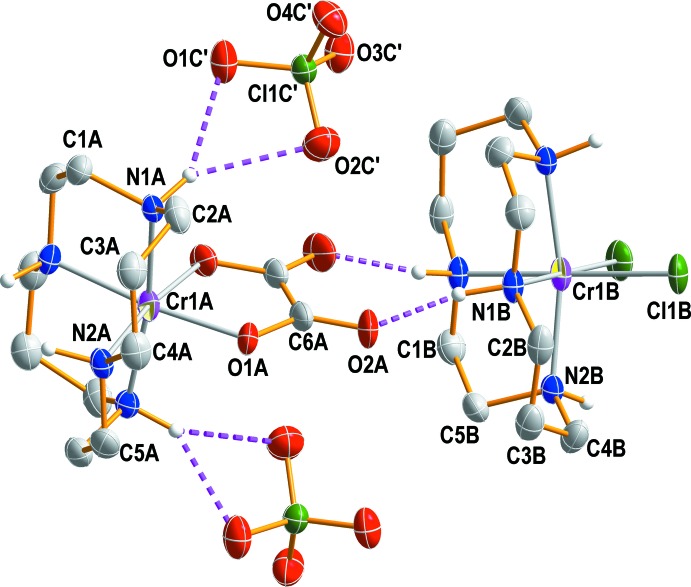
A perspective view of the two chromium(III) complex cations and two perchlorate anions in compound (I), drawn at the 30% probability level. The primed atoms are related by symmetry code (−*x* + 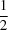, −*y* + 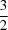, −*z*).

In the asymmetric unit, two N—H⋯O hydrogen bonds link the perchlorate anion to the neighboring [Cr(ox)(cyclam)]^+^ cation while N—H⋯O and N—H⋯Cl contacts inter­connect two [Cr(ox)(cyclam)]^+^ and one *cis-*[CrCl_2_(cyclam)]^+^ cation (Table 1 ▸, Figs. 2 ▸ and 3 ▸). An extensive array of these contacts generate a three-dimensional network of mol­ecules stacked along the *a*-axis direction.

## Database survey   

A search of the Cambridge Structural Database (Version 5.37, Feb 2016 with two updates; Groom *et al.*, 2016 ▸) gave 16 hits for a *cis*-[Cr*L*_2_(C_10_H_24_N_4_)]^+^ unit. The crystal structure of *cis-*[CrCl_2_(cyclam)]ClO_4_ (House & McKee, 1984 ▸), *cis-*[Cr(N_3_)_2_(cyclam)]ClO_4_ (Meyer *et al.*, 1998 ▸), *cis*-[Cr(NH_3_)_2_(cyclam)](ClO_4_)Cl_2_ (Derwahl *et al.*, 1999 ▸), *cis*-[Cr(ONO)_2_)(cyclam)]NO_2_ (Choi *et al.*, 2004*a* ▸), [Cr(ox)(cyclam)]ClO_4_ (ox = oxalate; Choi *et al.*, 2004*b* ▸), [Cr(acac)(cyclam)](ClO_4_)_2_·0.5H_2_O (acac = acetyl­acetonate; Subhan *et al.*, 2011 ▸) and *cis*-[Cr(NCS)_2_(cyclam)]NCS (Moon *et al.*, 2013 ▸) have been reported previously. All of these complexes show the same folded *cis*-V conformation for cyclam with different hydrogen-bonding and crystal-packing networks. Until now, no structure of the double complex ion [CrCl_2_(cyclam)][Cr(ox)(cyclam)]^2+^ with any anion has been deposited.

## Synthesis and crystallization   

The free ligand cyclam was purchased from Fluka and used as provided. All chemicals were reagent grade materials and were used without further purification. The starting materials, *cis-*[CrCl_2_(cyclam)]ClO_4_ and [Cr(ox)(cyclam)]ClO_4_, were prepared according to literature methods (House & McKee, 1984 ▸). The double complex, *cis-*[CrCl_2_(cyclam)][Cr(ox)(cyclam)](ClO_4_)_2_, was prepared by mixing concentrated equimolar aqueous solutions of the two starting compounds. A saturated solution of NaClO_4_ was added to the resulting solution for crystallization, and allowed to stand at room temperature for two days to give needle-like orange crystals of (I) suitable for X-ray structural analysis.

## Refinement   

Crystal data, data collection and structure refinement details are summarized in Table 2 ▸. Non-hydrogen atoms were refined anisotropically. All H atoms were placed in geometrically idealized positions and constrained to ride on their parent atoms, with C—H = 0.98 Å and N—H = 0.99 Å, and with *U*_iso_(H) values of 1.2*U*_eq_ of the parent atoms.

## Supplementary Material

Crystal structure: contains datablock(s) I. DOI: 10.1107/S2056989016014134/wm5323sup1.cif

Structure factors: contains datablock(s) I. DOI: 10.1107/S2056989016014134/wm5323Isup2.hkl

CCDC reference: 1502530

Additional supporting information: crystallographic information; 3D view; checkCIF report

## Figures and Tables

**Figure 3:**
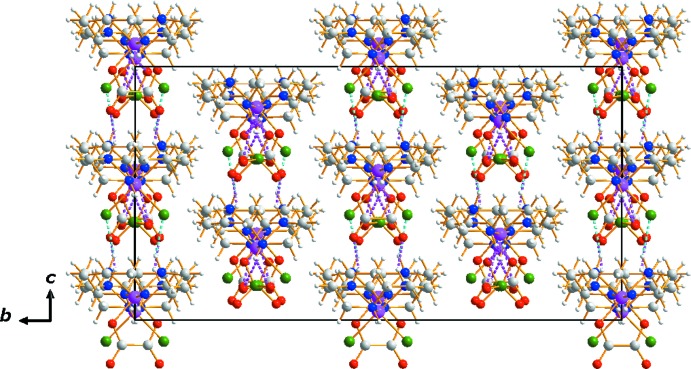
The crystal packing in compound (I), viewed perpendicular to the *bc* plane. Dashed lines represent N—H⋯O (pink) and N—H⋯Cl (cyan) hydrogen-bonding inter­actions, respectively.

**Table 1:** Hydrogen-bond geometry (Å, °)

*D*—H⋯*A*	*D*—H	H⋯*A*	*D*⋯*A*	*D*—H⋯*A*
N1*A*—H1*A*⋯O1*C* ^i^	0.99	2.20	3.090 (8)	148
N1*A*—H1*A*⋯O2*C* ^i^	0.99	2.42	3.266 (8)	143
N2*A*—H2*A*⋯Cl1*B* ^ii^	0.99	2.42	3.314 (5)	150
N1*B*—H1*B*⋯O2*A*	0.99	1.87	2.762 (7)	149
N2*B*—H2*B*⋯O4*C* ^iii^	0.99	2.39	3.160 (7)	135

Symmetry codes: (i) 
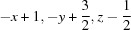
; (ii) 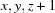; (iii) 
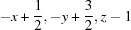
.

**Table 2:** Experimental details

Crystal data	
Chemical formula	[CrCl_2_(C_10_H_24_N_4_)][Cr(C_2_O_4_)(C_10_H_24_N_4_)](ClO_4_)_2_
*M*_r_	862.48
Crystal system, space group	Orthorhombic, *F* *d* *d*2
Temperature (K)	243
*a*, *b*, *c* (Å)	18.599 (4), 26.986 (5), 14.042 (3)
*V* (Å^3^)	7048 (2)
*Z*	8
Radiation type	Synchrotron, λ = 0.670 Å
μ (mm^−1^)	0.84
Crystal size (mm)	0.08 × 0.01 × 0.01

Data collection	
Diffractometer	ADSC Q210 CCD area detector
Absorption correction	Empirical (using intensity measurements) (*HKL3000sm *SCALEPACK**; Otwinowski & Minor, 1997 ▸)
*T*_min_, *T*_max_	0.939, 0.996
No. of measured, independent and observed [*I* > 2σ(*I*)] reflections	14619, 4764, 4011
*R*_int_	0.118
(sin θ/λ)_max_ (Å^−1^)	0.689

Refinement	
*R*[*F* ^2^ > 2σ(*F* ^2^)], *wR*(*F* ^2^), *S*	0.057, 0.150, 1.04
No. of reflections	4764
No. of parameters	218
No. of restraints	1
H-atom treatment	H-atom parameters constrained
Δρ_max_, Δρ_min_ (e Å^−3^)	1.54, −0.51
Absolute structure	Flack *x* determined using 1586 quotients [(*I* ^+^)−(*I* ^−^)]/[(*I* ^+^)+(*I* ^−^)] (Parsons *et al.*, 2013 ▸).
Absolute structure parameter	0.10 (2)

Computer programs: *PAL BL2D-SMDC* (Shin *et al.*, 2016 ▸), *HKL3000sm* (Otwinowski & Minor, 1997 ▸), *SHELXT2014* (Sheldrick, 2015*a* ▸), *SHELXL2014* (Sheldrick, 2015*b* ▸), *DIAMOND* (Putz & Brandenburg, 2014 ▸) and *publCIF* (Westrip, 2010 ▸).

## supplementary crystallographic information

### Crystal data

**Table:**

[CrCl_2_(C_10_H_24_N_4_)][Cr(C_2_O_4_)(C_10_H_24_N_4_)](ClO_4_)_2_	*D*_x_ = 1.626 Mg m^−^^3^
*M**_r_* = 862.48	Synchrotron radiation, λ = 0.670 Å
Orthorhombic, *F**d**d*2	Cell parameters from 25281 reflections
*a* = 18.599 (4) Å	θ = 0.4–33.3°
*b* = 26.986 (5) Å	µ = 0.84 mm^−^^1^
*c* = 14.042 (3) Å	*T* = 243 K
*V* = 7048 (2) Å^3^	Needle, orange
*Z* = 8	0.08 × 0.01 × 0.01 mm
*F*(000) = 3584	

### Data collection

**Table:**

ADSC Q210 CCD area detector diffractometer	4011 reflections with *I* > 2σ(*I*)
Radiation source: PLSII 2D bending magnet	*R*_int_ = 0.118
ω scan	θ_max_ = 27.5°, θ_min_ = 1.9°
Absorption correction: empirical (using intensity measurements) (*HKL3000sm Scalepack*; Otwinowski & Minor, 1997)	*h* = −25→25
*T*_min_ = 0.939, *T*_max_ = 0.996	*k* = −37→37
14619 measured reflections	*l* = −19→19
4764 independent reflections	

### Refinement

**Table:**

Refinement on *F*^2^	Hydrogen site location: inferred from neighbouring sites
Least-squares matrix: full	H-atom parameters constrained
*R*[*F*^2^ > 2σ(*F*^2^)] = 0.057	*w* = 1/[σ^2^(*F*_o_^2^) + (0.0928*P*)^2^] where *P* = (*F*_o_^2^ + 2*F*_c_^2^)/3
*wR*(*F*^2^) = 0.150	(Δ/σ)_max_ < 0.001
*S* = 1.04	Δρ_max_ = 1.54 e Å^−^^3^
4764 reflections	Δρ_min_ = −0.51 e Å^−^^3^
218 parameters	Absolute structure: Flack *x* determined using 1586 quotients [(*I*^+^)-(*I*^-^)]/[(*I*^+^)+(*I*^-^)] (Parsons *et al.*, 2013).
1 restraint	Absolute structure parameter: 0.10 (2)

### Special details

**Table:**

Geometry. All esds (except the esd in the dihedral angle between two l.s. planes) are estimated using the full covariance matrix. The cell esds are taken into account individually in the estimation of esds in distances, angles and torsion angles; correlations between esds in cell parameters are only used when they are defined by crystal symmetry. An approximate (isotropic) treatment of cell esds is used for estimating esds involving l.s. planes.

### Fractional atomic coordinates and isotropic or equivalent isotropic displacement parameters (Å^2^)

**Table:**

	*x*	*y*	*z*	*U*_iso_*/*U*_eq_	
Cr1A	0.2500	0.7500	0.83915 (6)	0.0342 (3)	
O1A	0.2305 (3)	0.70375 (18)	0.7351 (3)	0.0554 (10)	
O2A	0.2332 (4)	0.6997 (3)	0.5761 (4)	0.092 (2)	
N1A	0.3572 (3)	0.72957 (18)	0.8508 (3)	0.0457 (10)	
H1A	0.3813	0.7427	0.7932	0.055*	
N2A	0.2211 (3)	0.69512 (15)	0.9376 (3)	0.0426 (10)	
H2A	0.2340	0.7073	1.0018	0.051*	
C1A	0.3891 (4)	0.7568 (2)	0.9331 (5)	0.0557 (14)	
H1A1	0.4415	0.7581	0.9271	0.067*	
H1A2	0.3771	0.7400	0.9929	0.067*	
C2A	0.3745 (4)	0.6746 (2)	0.8539 (4)	0.0624 (17)	
H2A1	0.4263	0.6704	0.8636	0.075*	
H2A2	0.3622	0.6597	0.7923	0.075*	
C3A	0.3344 (4)	0.6470 (2)	0.9325 (5)	0.0603 (15)	
H3A1	0.3520	0.6128	0.9338	0.072*	
H3A2	0.3471	0.6622	0.9936	0.072*	
C4A	0.2546 (4)	0.6455 (2)	0.9255 (5)	0.0603 (17)	
H4A1	0.2410	0.6321	0.8631	0.072*	
H4A2	0.2357	0.6231	0.9744	0.072*	
C5A	0.1417 (4)	0.6917 (2)	0.9327 (5)	0.0587 (15)	
H5A1	0.1234	0.6730	0.9874	0.070*	
H5A2	0.1273	0.6744	0.8744	0.070*	
C6A	0.2399 (3)	0.7232 (3)	0.6505 (4)	0.0637 (18)	
Cr1B	0.2500	0.7500	0.28338 (5)	0.0319 (3)	
Cl1B	0.26881 (11)	0.69068 (6)	0.16485 (9)	0.0602 (4)	
N1B	0.2679 (3)	0.69487 (15)	0.3851 (3)	0.0430 (10)	
H1B	0.2563	0.7094	0.4480	0.052*	
N2B	0.1403 (3)	0.73775 (18)	0.2995 (3)	0.0446 (9)	
H2B	0.1173	0.7524	0.2425	0.054*	
C1B	0.3458 (4)	0.6843 (2)	0.3845 (5)	0.0554 (14)	
H1B1	0.3582	0.6643	0.3287	0.066*	
H1B2	0.3591	0.6657	0.4418	0.066*	
C2B	0.2277 (4)	0.64797 (19)	0.3775 (4)	0.0558 (15)	
H2B1	0.2429	0.6256	0.4287	0.067*	
H2B2	0.2392	0.6321	0.3166	0.067*	
C3B	0.1470 (4)	0.6558 (2)	0.3838 (5)	0.0629 (17)	
H3B1	0.1235	0.6233	0.3855	0.076*	
H3B2	0.1362	0.6726	0.4439	0.076*	
C4B	0.1151 (4)	0.6856 (3)	0.3030 (4)	0.0633 (17)	
H4B1	0.1271	0.6694	0.2426	0.076*	
H4B2	0.0626	0.6854	0.3093	0.076*	
C5B	0.1140 (4)	0.7675 (2)	0.3819 (5)	0.0567 (13)	
H5B1	0.1218	0.7493	0.4413	0.068*	
H5B2	0.0623	0.7738	0.3752	0.068*	
Cl1C	0.52532 (8)	0.74678 (4)	1.13543 (9)	0.0464 (3)	
O1C	0.5216 (4)	0.7246 (2)	1.2288 (3)	0.0738 (15)	
O2C	0.5875 (3)	0.7782 (2)	1.1306 (5)	0.0812 (15)	
O3C	0.5278 (3)	0.70980 (16)	1.0630 (3)	0.0666 (13)	
O4C	0.4626 (3)	0.7775 (2)	1.1228 (4)	0.0696 (13)	

### Atomic displacement parameters (Å^2^)

**Table:**

	*U*^11^	*U*^22^	*U*^33^	*U*^12^	*U*^13^	*U*^23^
Cr1A	0.0410 (6)	0.0458 (5)	0.0159 (4)	−0.0046 (4)	0.000	0.000
O1A	0.064 (3)	0.072 (3)	0.0296 (17)	−0.009 (2)	−0.0016 (18)	−0.0161 (18)
O2A	0.091 (4)	0.153 (5)	0.031 (2)	−0.018 (4)	0.004 (2)	−0.032 (3)
N1A	0.043 (2)	0.067 (3)	0.0267 (16)	0.000 (2)	0.0034 (16)	−0.0047 (17)
N2A	0.057 (3)	0.0408 (18)	0.0300 (18)	−0.0026 (18)	0.0062 (18)	0.0020 (14)
C1A	0.052 (4)	0.076 (4)	0.039 (3)	−0.002 (3)	−0.007 (2)	−0.004 (2)
C2A	0.071 (5)	0.075 (4)	0.042 (3)	0.023 (3)	0.004 (3)	−0.007 (3)
C3A	0.077 (4)	0.050 (2)	0.054 (3)	0.014 (3)	−0.001 (3)	−0.004 (2)
C4A	0.087 (5)	0.039 (2)	0.055 (3)	0.005 (2)	0.005 (3)	0.001 (2)
C5A	0.065 (4)	0.064 (3)	0.047 (3)	−0.020 (3)	0.010 (3)	0.004 (2)
C6A	0.047 (3)	0.119 (6)	0.025 (2)	−0.006 (3)	0.0010 (19)	−0.004 (3)
Cr1B	0.0459 (6)	0.0333 (4)	0.0164 (4)	0.0007 (4)	0.000	0.000
Cl1B	0.0926 (11)	0.0583 (7)	0.0297 (6)	0.0046 (7)	0.0025 (6)	−0.0161 (5)
N1B	0.069 (3)	0.0350 (17)	0.0251 (17)	0.0053 (19)	0.0005 (19)	0.0000 (14)
N2B	0.047 (2)	0.062 (2)	0.0255 (18)	0.000 (2)	−0.0011 (15)	0.0011 (17)
C1B	0.064 (4)	0.053 (3)	0.049 (3)	0.014 (3)	−0.003 (3)	0.003 (2)
C2B	0.098 (5)	0.0337 (19)	0.036 (2)	−0.006 (3)	−0.002 (3)	0.0030 (17)
C3B	0.090 (5)	0.055 (3)	0.044 (3)	−0.020 (3)	−0.002 (3)	0.004 (2)
C4B	0.077 (5)	0.072 (3)	0.041 (3)	−0.026 (3)	−0.009 (3)	0.000 (3)
C5B	0.052 (3)	0.076 (3)	0.041 (3)	0.001 (3)	0.011 (3)	−0.006 (3)
Cl1C	0.0524 (8)	0.0494 (6)	0.0372 (6)	0.0003 (5)	−0.0027 (5)	−0.0003 (5)
O1C	0.109 (5)	0.073 (3)	0.040 (2)	0.002 (3)	−0.001 (3)	0.004 (2)
O2C	0.081 (4)	0.084 (3)	0.079 (3)	−0.023 (3)	0.003 (3)	−0.003 (3)
O3C	0.100 (4)	0.053 (2)	0.046 (2)	0.005 (2)	0.009 (2)	−0.0049 (18)
O4C	0.070 (3)	0.077 (3)	0.062 (3)	0.020 (3)	−0.011 (2)	−0.012 (2)

### Geometric parameters (Å, º)

**Table:**

Cr1A—O1A^i^	1.956 (4)	Cr1B—N2B	2.080 (5)
Cr1A—O1A	1.956 (4)	Cr1B—N1B^i^	2.089 (4)
Cr1A—N1A^i^	2.075 (5)	Cr1B—N1B	2.089 (4)
Cr1A—N1A	2.075 (5)	Cr1B—Cl1B	2.3358 (14)
Cr1A—N2A	2.096 (4)	Cr1B—Cl1B^i^	2.3358 (14)
Cr1A—N2A^i^	2.096 (4)	N1B—C2B	1.474 (7)
O1A—C6A	1.310 (8)	N1B—C1B	1.478 (9)
O2A—C6A	1.228 (8)	N1B—H1B	0.9900
N1A—C1A	1.493 (8)	N2B—C4B	1.484 (8)
N1A—C2A	1.519 (8)	N2B—C5B	1.490 (7)
N1A—H1A	0.9900	N2B—H2B	0.9900
N2A—C5A	1.482 (9)	C1B—C5B^i^	1.500 (9)
N2A—C4A	1.485 (7)	C1B—H1B1	0.9800
N2A—H2A	0.9900	C1B—H1B2	0.9800
C1A—C5A^i^	1.502 (10)	C2B—C3B	1.518 (11)
C1A—H1A1	0.9800	C2B—H2B1	0.9800
C1A—H1A2	0.9800	C2B—H2B2	0.9800
C2A—C3A	1.526 (10)	C3B—C4B	1.512 (9)
C2A—H2A1	0.9800	C3B—H3B1	0.9800
C2A—H2A2	0.9800	C3B—H3B2	0.9800
C3A—C4A	1.488 (11)	C4B—H4B1	0.9800
C3A—H3A1	0.9800	C4B—H4B2	0.9800
C3A—H3A2	0.9800	C5B—C1B^i^	1.500 (9)
C4A—H4A1	0.9800	C5B—H5B1	0.9800
C4A—H4A2	0.9800	C5B—H5B2	0.9800
C5A—C1A^i^	1.503 (10)	Cl1C—O3C	1.426 (5)
C5A—H5A1	0.9800	Cl1C—O2C	1.435 (6)
C5A—H5A2	0.9800	Cl1C—O4C	1.442 (5)
C6A—C6A^i^	1.496 (18)	Cl1C—O1C	1.443 (5)
Cr1B—N2B^i^	2.080 (5)		
			
O1A^i^—Cr1A—O1A	83.3 (3)	N2B^i^—Cr1B—N1B^i^	88.2 (2)
O1A^i^—Cr1A—N1A^i^	93.85 (19)	N2B—Cr1B—N1B^i^	83.26 (19)
O1A—Cr1A—N1A^i^	92.9 (2)	N2B^i^—Cr1B—N1B	83.26 (19)
O1A^i^—Cr1A—N1A	92.9 (2)	N2B—Cr1B—N1B	88.2 (2)
O1A—Cr1A—N1A	93.85 (19)	N1B^i^—Cr1B—N1B	93.7 (2)
N1A^i^—Cr1A—N1A	171.0 (2)	N2B^i^—Cr1B—Cl1B	92.27 (13)
O1A^i^—Cr1A—N2A	172.4 (2)	N2B—Cr1B—Cl1B	96.65 (14)
O1A—Cr1A—N2A	89.67 (18)	N1B^i^—Cr1B—Cl1B	177.69 (13)
N1A^i^—Cr1A—N2A	83.68 (19)	N1B—Cr1B—Cl1B	88.58 (12)
N1A—Cr1A—N2A	90.38 (19)	N2B^i^—Cr1B—Cl1B^i^	96.65 (14)
O1A^i^—Cr1A—N2A^i^	89.67 (18)	N2B—Cr1B—Cl1B^i^	92.27 (13)
O1A—Cr1A—N2A^i^	172.4 (2)	N1B^i^—Cr1B—Cl1B^i^	88.58 (12)
N1A^i^—Cr1A—N2A^i^	90.37 (19)	N1B—Cr1B—Cl1B^i^	177.69 (13)
N1A—Cr1A—N2A^i^	83.68 (19)	Cl1B—Cr1B—Cl1B^i^	89.11 (9)
N2A—Cr1A—N2A^i^	97.5 (2)	C2B—N1B—C1B	109.4 (5)
C6A—O1A—Cr1A	113.4 (5)	C2B—N1B—Cr1B	118.8 (4)
C1A—N1A—C2A	112.0 (5)	C1B—N1B—Cr1B	106.8 (3)
C1A—N1A—Cr1A	108.2 (4)	C2B—N1B—H1B	107.1
C2A—N1A—Cr1A	117.7 (4)	C1B—N1B—H1B	107.1
C1A—N1A—H1A	106.1	Cr1B—N1B—H1B	107.1
C2A—N1A—H1A	106.1	C4B—N2B—C5B	112.5 (5)
Cr1A—N1A—H1A	106.1	C4B—N2B—Cr1B	117.6 (4)
C5A—N2A—C4A	110.9 (5)	C5B—N2B—Cr1B	108.7 (4)
C5A—N2A—Cr1A	105.6 (4)	C4B—N2B—H2B	105.7
C4A—N2A—Cr1A	117.0 (4)	C5B—N2B—H2B	105.7
C5A—N2A—H2A	107.7	Cr1B—N2B—H2B	105.7
C4A—N2A—H2A	107.7	N1B—C1B—C5B^i^	108.8 (5)
Cr1A—N2A—H2A	107.7	N1B—C1B—H1B1	109.9
N1A—C1A—C5A^i^	107.5 (5)	C5B^i^—C1B—H1B1	109.9
N1A—C1A—H1A1	110.2	N1B—C1B—H1B2	109.9
C5A^i^—C1A—H1A1	110.2	C5B^i^—C1B—H1B2	109.9
N1A—C1A—H1A2	110.2	H1B1—C1B—H1B2	108.3
C5A^i^—C1A—H1A2	110.2	N1B—C2B—C3B	112.2 (5)
H1A1—C1A—H1A2	108.5	N1B—C2B—H2B1	109.2
N1A—C2A—C3A	113.2 (5)	C3B—C2B—H2B1	109.2
N1A—C2A—H2A1	108.9	N1B—C2B—H2B2	109.2
C3A—C2A—H2A1	108.9	C3B—C2B—H2B2	109.2
N1A—C2A—H2A2	108.9	H2B1—C2B—H2B2	107.9
C3A—C2A—H2A2	108.9	C4B—C3B—C2B	114.8 (6)
H2A1—C2A—H2A2	107.7	C4B—C3B—H3B1	108.6
C4A—C3A—C2A	116.9 (6)	C2B—C3B—H3B1	108.6
C4A—C3A—H3A1	108.1	C4B—C3B—H3B2	108.6
C2A—C3A—H3A1	108.1	C2B—C3B—H3B2	108.6
C4A—C3A—H3A2	108.1	H3B1—C3B—H3B2	107.6
C2A—C3A—H3A2	108.1	N2B—C4B—C3B	113.9 (5)
H3A1—C3A—H3A2	107.3	N2B—C4B—H4B1	108.8
N2A—C4A—C3A	112.7 (5)	C3B—C4B—H4B1	108.8
N2A—C4A—H4A1	109.0	N2B—C4B—H4B2	108.8
C3A—C4A—H4A1	109.0	C3B—C4B—H4B2	108.8
N2A—C4A—H4A2	109.0	H4B1—C4B—H4B2	107.7
C3A—C4A—H4A2	109.0	N2B—C5B—C1B^i^	108.8 (5)
H4A1—C4A—H4A2	107.8	N2B—C5B—H5B1	109.9
N2A—C5A—C1A^i^	108.8 (5)	C1B^i^—C5B—H5B1	109.9
N2A—C5A—H5A1	109.9	N2B—C5B—H5B2	109.9
C1A^i^—C5A—H5A1	109.9	C1B^i^—C5B—H5B2	109.9
N2A—C5A—H5A2	109.9	H5B1—C5B—H5B2	108.3
C1A^i^—C5A—H5A2	109.9	O3C—Cl1C—O2C	110.7 (4)
H5A1—C5A—H5A2	108.3	O3C—Cl1C—O4C	110.0 (3)
O2A—C6A—O1A	123.4 (8)	O2C—Cl1C—O4C	107.8 (4)
O2A—C6A—C6A^i^	121.7 (5)	O3C—Cl1C—O1C	111.0 (3)
O1A—C6A—C6A^i^	114.9 (4)	O2C—Cl1C—O1C	109.1 (4)
N2B^i^—Cr1B—N2B	167.5 (2)	O4C—Cl1C—O1C	108.1 (3)
			
C2A—N1A—C1A—C5A^i^	−170.2 (5)	Cr1A—O1A—C6A—C6A^i^	−2.7 (9)
Cr1A—N1A—C1A—C5A^i^	−38.9 (6)	C2B—N1B—C1B—C5B^i^	174.1 (5)
C1A—N1A—C2A—C3A	71.3 (7)	Cr1B—N1B—C1B—C5B^i^	44.3 (5)
Cr1A—N1A—C2A—C3A	−55.0 (6)	C1B—N1B—C2B—C3B	176.5 (5)
N1A—C2A—C3A—C4A	63.5 (7)	Cr1B—N1B—C2B—C3B	−60.6 (6)
C5A—N2A—C4A—C3A	−178.5 (5)	N1B—C2B—C3B—C4B	64.6 (6)
Cr1A—N2A—C4A—C3A	60.4 (7)	C5B—N2B—C4B—C3B	−67.7 (8)
C2A—C3A—C4A—N2A	−66.6 (7)	Cr1B—N2B—C4B—C3B	59.8 (7)
C4A—N2A—C5A—C1A^i^	−173.1 (5)	C2B—C3B—C4B—N2B	−64.9 (8)
Cr1A—N2A—C5A—C1A^i^	−45.5 (5)	C4B—N2B—C5B—C1B^i^	167.8 (6)
Cr1A—O1A—C6A—O2A	177.1 (6)	Cr1B—N2B—C5B—C1B^i^	35.7 (6)

Symmetry code: (i) −*x*+1/2, −*y*+3/2, *z*.

### Hydrogen-bond geometry (Å, º)

**Table:**

*D*—H···*A*	*D*—H	H···*A*	*D*···*A*	*D*—H···*A*
N1*A*—H1*A*···O1*C*^ii^	0.99	2.20	3.090 (8)	148
N1*A*—H1*A*···O2*C*^ii^	0.99	2.42	3.266 (8)	143
N2*A*—H2*A*···Cl1*B*^iii^	0.99	2.42	3.314 (5)	150
N1*B*—H1*B*···O2*A*	0.99	1.87	2.762 (7)	149
N2*B*—H2*B*···O4*C*^iv^	0.99	2.39	3.160 (7)	135

Symmetry codes: (ii) −*x*+1, −*y*+3/2, *z*−1/2; (iii) *x*, *y*, *z*+1; (iv) −*x*+1/2, −*y*+3/2, *z*−1.
